# An Activated Form of UFO Alters Leaf Development and Produces Ectopic Floral and Inflorescence Meristems

**DOI:** 10.1371/journal.pone.0083807

**Published:** 2013-12-23

**Authors:** Eddy Risseeuw, Prakash Venglat, Daoquan Xiang, Kristina Komendant, Tim Daskalchuk, Vivijan Babic, William Crosby, Raju Datla

**Affiliations:** 1 Plant Biotechnology Institute, National Research Council, Saskatoon, Canada; 2 Department of Biological Sciences, University of Windsor, Windsor, Canada; Ohio State University, United States of America

## Abstract

Plants are unique in their ability to continuously produce new meristems and organ primordia. In Arabidopsis, the transcription factor LEAFY (LFY) functions as a master regulator of a gene network that is important for floral meristem and organ specification. UNUSUAL FLORAL ORGANS (UFO) is a co-activator of LEAFY and is required for proper activation of *APETALA3* in the floral meristem during the specification of stamens and petals. The *ufo* mutants display defects in other parts of the flower and the inflorescence, suggestive of additional roles. Here we show that the normal determinacy of the developing Arabidopsis leaves is affected by the expression of a gain-of-function UFO fusion protein with the VP16 transcriptional activator domain. In these lines, the rosette and cauline leaf primordia exhibit reiterated serration, and upon flowering produce ectopic meristems that develop into flowers, bract leaves and inflorescences. These striking phenotypes reveal that developing leaves maintain the competency to initiate flower and inflorescence programs. Furthermore, the gain-of-function phenotypes are dependent on LFY and the SEPALLATA (SEP) MADS-box transcription factors, indicative of their functional interactions with UFO. The findings of this study also suggest that UFO promotes the establishment of the lateral meristems and primordia in the peripheral zone of the apical and floral meristems by enhancing the activity of LFY. These novel phenotypes along with the mutant phenotypes of UFO orthologs in other plant species suggest a broader function for UFO in plants.

## Introduction

The continuous production of new meristems is a characteristic feature in plants and accounts for their distinctive indeterminate growth. After germination, the shoot apical meristem (SAM) produces phytomers repetitively which represent an internodal stem with a node comprising of a leaf subtending an axillary meristem [1]. The apical and axillary meristems are usually indeterminate [2], whereas the leaf primordia are most often determinate [3]. During the course of its life cycle, the plant produces multiple meristems and primordia and these will acquire different identities to give rise to different organs. The meristems can switch identity over time and this is particularly important during the transition from the vegetative to the reproductive phase. The identities are determined by the combinatorial expression and functions of specific meristem and organ identity genes controlled spatially and temporally by preprogrammed genetic networks [4]. In Arabidopsis, the LEAFY (LFY) protein is a master regulator of the organ identity genes and its function is essential for both conferring floral meristem identity and the subsequent identity of the individual floral organs. *LFY* is a plant specific transcription factor and activates several key floral organ identity genes including the ABC class MADS-box genes [5]. The *lfy* mutant is impaired in the floral fate specification of the meristems produced by the inflorescence meristem, and as a consequence, new meristems default towards a co-inflorescence fate, resulting in a leafy appearance [6], [7].

UNUSUAL FLORAL ORGANS (UFO) is a key cofactor of LFY to specify the petal and stamen whorls by regulating the expression of the B-class MADS-box gene *APETALA 3* (AP3) in the floral meristem [8], [9], [10]. Therefore in *ufo* flowers, petal and stamen development is severely affected resulting in either reduction or complete absence of these organs [11]. The *ufo* mutant is also associated with a range of additional defects outside the *AP3* expression domain including the loss of carpels in some flowers; replacement of flowers by filaments; and perturbed transition of the apical meristem from the vegetative to the inflorescence identity, particularly under short day conditions [12]. These findings suggest that the spatial and temporal overlap of the *LFY* and *UFO* expression domains are important for specification of the floral meristem and the floral organ primordia [13]. Compared to Arabidopsis, the expression domains of the *LFY* and *UFO* orthologs vary considerably in other plant species [14]. Accordingly their individual functions and their respective mutant phenotypes are quite different from the Arabidopsis *lfy* and *ufo* mutants. For example, the *UFO* ortholog *double top* (*dot*) mutant in petunia is unable to produce flowers, whereas in the pea *unifoliata (uni)* and s*tamina pistilloida* (*stp*) mutants, the orthologs of *LFY* and *UFO*, show reduced leaf complexity in addition to inflorescence abnormalities [15], [16]. Though *ufo* mutants do not affect the leaf shape in Arabidopsis, the ectopic expression of *UFO* results in serrated leaves [8]. These phenotypes indicate that the function of *UFO* and its homologs in other species are not restricted only to the specification of the petal and stamen whorls, but also extended to include other meristems and primordia both inside and outside of the floral program.

UFO belongs to a large group of F-box proteins encoded by a family of over 700 genes in Arabidopsis [17], [18]. F-box proteins confer specificity to the SCF (Skp1-cullin-F-box complex) class of ubiquitin ligases by binding and presenting specific target proteins to the ubiquitin conjugating enzyme [19]. The UFO F-box domain is required for the interaction with the Skp1 adapter protein (ASK proteins in Arabidopsis) and the COP9 signalosome [17], [20]. The C-terminal domain of UFO has been shown to bind LFY and this interaction likely leads to its ubiquitination [10], [15]. Transcription factors are often substrates for ubiquitination where this modification plays a dual role by activation followed by their turnover [21], [22], [23], [24]. Therefore, UFO plays a unique role in flower development by ubiquitinating the plant specific transcription factor, LFY, which likely also requires the participation of UFO in the transcriptional complex [10]. In this study, the involvement of UFO in a transcriptional complex was tested by the construction of activator and repressor versions of UFO employing the well characterized and widely used heterologous VP16 based activator and Engrailed based repressor domains respectively. The developmental effects produced by these overexpression constructs were evaluated in transgenic Arabidopsis, *Brassica napus* and tobacco plants. Our results showed that especially the activator fusion had a dramatic effect on the UFO gain-of-function phenotypes that include development of ectopic flowers and inflorescences subtended by bracts on Arabidopsis leaves. Analysis of these novel leaf phenotypes revealed potential functions of UFO outside the flower context. Additionally, comparison of *UFO* functions with its orthologs in other plant species also suggests a broader role for *UFO* during meristem establishment and specification.

## Results

### UFO is a transcriptional co-activator

**Figure 1:**
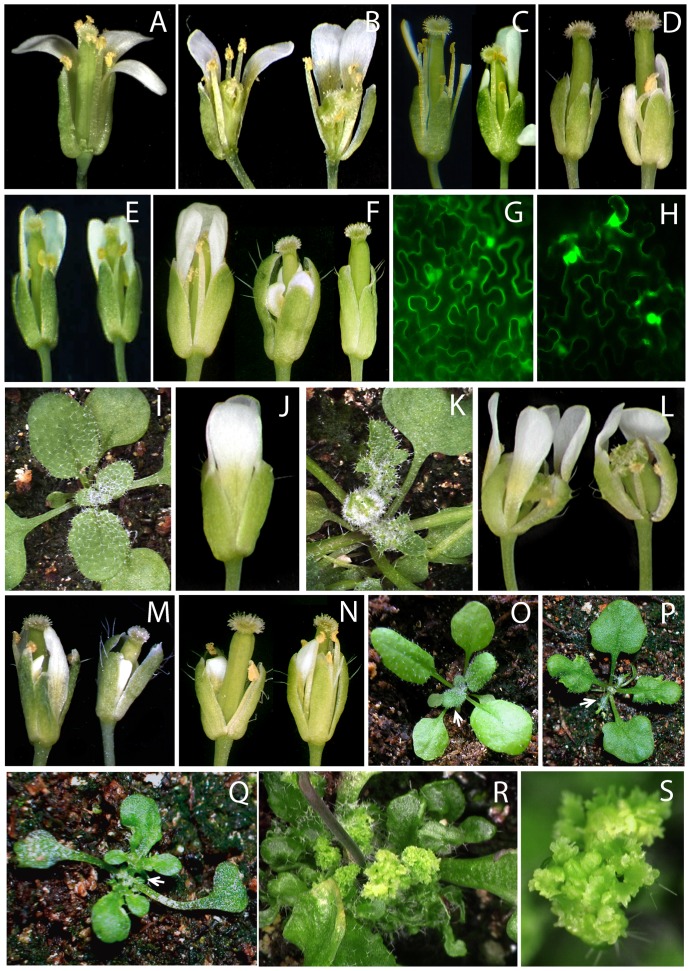
Phenotypes of Arabidopsis lines overexpressing UFO and its variants. (A-L) Interaction with ASK1 is required for the nuclear function of UFO. (A–F) Phenotypes of Arabidopsis flowers from WT (A) compared with flowers from the following transgenic lines; *p35S:UFO* showing abnormal development of the gynoecium and the style (B); *ask1-1* (C) and *ufo-1* (D) showing fewer stamens; *ask1-1 p35S: UFO* showing normal gynoecium development (E); *p35S:UFOdelF* with weak, medium and strong *ufo-1* mutant-like phenotypes (F). (G, H) Transient expression of *p35S:YFP* (G) and *p35S:UFO-YFP* (H) in *Nicotiana benthamiana* epidermal cells. Accumulation of UFO-YFP in the nucleus; (I–L) Respective uninduced controls (I,J) and dexamethasone induced (K,L) Arabidopsis plants and their flowers expressing UFO-GR. Nuclear translocation of the UFO glucocorticoid receptor fusion in the presence of dexamethasone resulted in leaf serration and floral defects reminiscent of the plants transformed with *p35S:UFO* (B). (M–S) UFO fusions with the *engrailed* (*En*) transcriptional repressor domain and with the VP16 transactivator domain. (M) *p35S:En-UFO* and (N) *p35S:En-UFOdelF* flowers showing strong phenotype. (O–Q) 3 week-old rosette plants of WT (O), *p35S:UFO-VP16* showing medium (P) and strong (Q) phenotypes. Arrows indicate delayed leaf expansion. (R) *p35S:UFO-VP16* in *sgs2-1* mutant background showing strong phenotype with proliferative rosette leaves (R). A close up of the ectopic floral organs formed on the leaf tip is shown in (S).

To assess the functions of UFO, Arabidopsis plants were transformed with the *UFO* gene under the control of the cauliflower mosaic virus 35S (*CaMV 35S*) promoter. 22% of the transgenic plants produced serrate leaves and flowers with reduced sepals and abnormalities in the development of the gynoecium valves and the style. Overall these plants displayed relatively milder phenotypes than the *35S:UFO* phenotypes reported by Lee et al (Table S1; Figure 1B) [8]. Expression of *UFO* under the control of the *LFY* promoter also resulted in serrated leaves, but occurred less frequently among the T1 transgenic lines (4%) compared to the expression with the 35S promoter (22%), suggesting that higher UFO levels were also required for the overexpression phenotypes (Table S1). When *UFO* was over-expressed in the *sgs2-1* background, which suppresses gene silencing [25], all T1 plants showed the gain-of-function phenotypes with enhanced leaf serration (Table S1). These results suggest that the *p35S:UFO* plants (78%) with a weak and medium loss-of-function phenotypes in the wild type background were the result of partial silencing of the endogenous *UFO* gene and that high UFO levels were required for the observed leaf serration and other phenotypic changes (Table S1).

Flowers of the *ask1-1* mutant showed fewer petals compared to wild type and were often replaced with petal-stamen chimeras, which resemble the flowers of an intermediate *ufo-1* mutant implying a genetic interaction between these two genes (Figure 1C, D) [12], [26]. Accordingly, the interaction of UFO with the most abundant ASK1 protein [18] was required for the *35S:UFO* phenotype because the *ask1-1* mutation was epistatic to the *UFO* transgene (Figure 1E) and over-expression of *UFO* carrying a mutation in the F-box resulted in a weak *ufo* dominant-negative phenotype (Figure 1F). These phenotypes are also consistent with the paradoxical “activation by destruction” model which has been implicated for LFY [10]. This model predicts that ubiquitination of transcription factors results in simultaneous activation and priming for destruction. Thus, UFO lacking the F-box domain is expected to stabilize un-ubiquitinated LFY and thereby repressing its activity. UFO is predominantly a nuclear protein as shown by the accumulation of a UFO-YFP fusion in the nucleus upon transient expression in *Nicotiana benthamiana* leaves (Figure 1G, H). This result also confirmed that translocation of UFO into the nucleus is required for its activity because fusion to the glucocorticoid receptor (GR), which is known to be retained in the cytosol in the absence of hormone, is unable to induce a gain-of-function phenotypes (Table S1; Figure 1 I, J). As expected, dexamethasone (DEX) induced translocation of UFO-GR from the cytosol into the nucleus, correlated with the initiation of serrated leaves and abnormal flowers in 23% of the transgenic lines (Table S1; Figure 1K, L). Taken together, the results showed that the function of UFO depends on its nuclear localization and its interaction with ASK1.

LFY is a key transcription factor in flower development and has been identified as a likely substrate for ubiquitination by UFO [10]. Because UFO does not contain any known DNA binding domains, we reasoned that UFO likely functions in the context of a transcriptional complex that includes LFY. In this situation, the fusion of UFO to the Drosophila Engrailed (En) transcriptional repressor domain or to the viral VP16 activator domain was expected to influence the function of this putative transcriptional complex in a dominant negative (loss-of-function) and a dominant (gain-of-function) manner respectively. This was indeed the case and the strong novel phenotypes identified in this study further indicate potential additional roles for UFO in plant development.

When the En-UFO translational fusion was over-expressed in Arabidopsis under the *35S* promoter, ∼85% of the T1 plants exhibited a range of mild to strong *ufo-1* like phenotypes with normal leaves. Overall these phenotypes were stronger compared to the plants expressing UFO without the F-box, but weaker than the reported UFO-SRDX fusion (Table S1; Figure 1M) [10]. Upon combining the Engrailed domain with the F-box deletion, more T1 transgenic plants showed strong loss-of-function phenotypes with flowers containing sepal-petal, stamen-carpel chimera or filaments in the second and third whorl or flower-filament substitutions in the inflorescence (Table S1; Figure 1N). In contrast, every T1 plant expressing the UFO-VP16 fusion exhibited leaf serration (Figure 1P, Q), which was more severe compared to plants over-expressing UFO only. RT-PCR analysis revealed an 8-fold increase in the *UFO-VP16* transcript levels compared to endogenous *UFO* RNA consistent with the observed phenotypes (Figure S1; see Methods). Based on the onset and severity of the leaf serration, the transgenic lines could be divided into two groups. About 86% of the plants showed clear serration starting in the third or fourth true leaf and these were designated as the weak and medium phenotype group whereas the plants in the strong phenotype group showed serration of all rosette leaves (Table S1; Figure 1P, Q). Compared to wild type and *35S:UFO* transgenic plants, the emergence of the first true leaves was delayed in *UFO-VP16* expressing lines, especially in the plants that showed strong gain-of-function phenotypes. The serrations became increasingly severe in later formed leaves. When *UFO-VP16* was expressed in the *sgs2-1* background, all T1 plants from the weak and medium phenotype group displayed a strong gain-of function phenotype, indicating that the weaker phenotype was caused by either lower UFO-VP16 protein expression and/or by partial silencing of the endogenous *UFO* gene (Table S1; Figure 1R, S). The result that the *VP16* transcriptional activator and not the *En* repressor enhanced the *UFO* over-expression phenotype suggests that *UFO* likely functions as a transcriptional co-activator.

### UFO-VP16 causes flowering leaves

**Figure 2:**
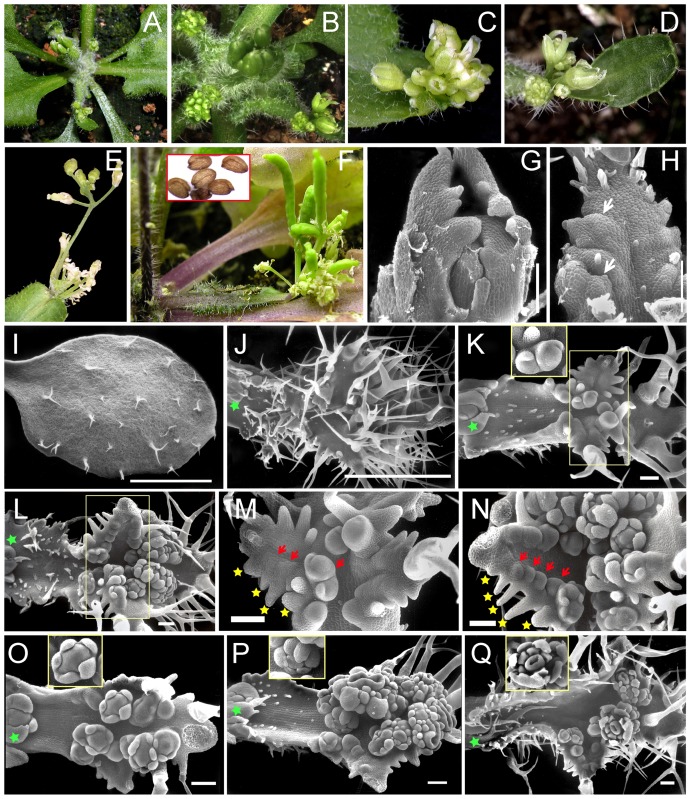
Characterization of ectopic flowers produced on the rosette leaves of *p35S:UFO-VP16* Arabidopsis plants with a medium phenotype. (A–D) Upper rosette leaves of *p35S:UFO-VP16* plant (A) with ectopic flowers (B; C-close-up), occasionally subtended by an ectopic bract (D). (E–F) Ectopic inflorescences on the rosette leaves of *p35S:UFO-VP16* plants (E) that produce siliques (F) and fertile seeds upon pollination (inset in F). (G–N) Ontogeny of the ectopic flower/inflorescence formation on the leaves of *p35S:UFO-VP16* plants. Developing lower rosette leaf primordia showing serrations along the margins (G) that produce secondary serrations (arrows) at a later stage (H). Developing upper rosette leaves of WT (I) and *p35S:UFO-VP16* (J–Q); rosette leaf prior to flowering showing deep serrations and an excess of enlarged trichomes (J); serrated rosette leaves formed at flowering with ectopic floral/inflorescence meristems (K–Q) that show progressive floral developmental stages (insets in K, O–Q). Grey boxed regions in K and L are magnified in M and N; red arrows indicate the emerging floral primordia that are positioned at distal ends of the serrations (yellow stars). Green stars in J–L, O–Q indicate axillary meristems. Bar = 1 mm (I, J); 0.1 mm (G, H, K–Q).

Overexpression of UFO fused to the VP16 transactivation domain resulted in unexpected phenotypes not observed in the earlier studies. 50% of the T1 plants, which belonged to the first group developed flowers along the adaxial edge beginning in the last three rosette leaves that are formed prior to bolting and later in all the cauline leaves (Figure 2A–C). These floral meristems were ectopic because they were neither derived from the apical meristem nor from the displacement of the axillary meristem. Although most of the leaf blade originated from the basal part of the leaf, the flowering rosette leaves often grew to a normal size. Sometimes the flowers were also subtended by a bract leaf (Figure 2D). Occasionally an inflorescence would develop in addition to the individual flowers (Figure 2E). These flowers were mostly male sterile, but produced viable seeds upon pollination (Figure 2F). Closer examination revealed that the lower rosette leaf primordia had serrations along their margins that became compounded at later stages as shown by secondary serrations (Figure 2G, H). These leaf primordia exhibited a prolonged primordial phase and eventually developed into severely lobed leaves that were covered with enlarged trichomes (Figure 2J). The upper rosette leaf primordia that were formed during floral transition were also serrated and initiated floral meristems that developed in a sequential acropetal fashion along the adaxial margins (Figure 2K–N). It is interesting to note that each developing floral meristem (indicated by red arrow in Figure 2M, N) was positioned at the distal end of a serration (indicated by yellow star in Figure 2M, N), and few of them formed bract-like structures (Figure 2D). These ectopic floral meristems followed the normal developmental progression as defined by the stages shown in the insets of Figures 2K,O–Q [27]. The floral meristems on the leaves developed into complete flowers including the pedicel, and were similar to the flowers produced on the main inflorescence. Often these ectopic floral meristems were fused to produce fasciations of the pedicel or of the flowers themselves. Few of these ectopic meristems developed into inflorescence meristems that produced flowers along their flanks (Figure 2M, 2E). These phenotypes indicate that the inflorescence program, which is normally established in the axis of the leaf, now expanded into the leaf primordium during its extended meristematic phase.

**Figure 3:**
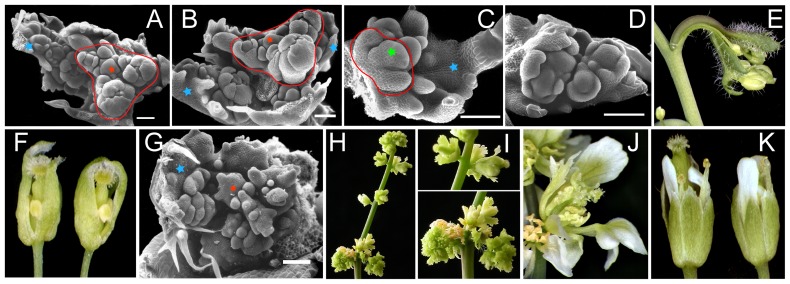
Inflorescence phenotypes of *p35S:UFO-VP16* Arabidopsis plants. (A–F) *p35S:UFO-VP16* inflorescence with a medium phenotype showing the developing flowers (outlined in red) produced by the primary inflorescence meristem (red star) (A - top view, B - side view) and by the cauline leaves (blue star) shown at different developmental stages (A–E); the developing co-florescence meristem (green star) and the transformed cauline leaf primordium (C) fail to separate leading to a fasciated structure (E); flowers show reduced and short petals, stamens and carpels (F). (G–J) *p35S:UFO-VP16* inflorescence with a strong phenotype showing an disordered inflorescence meristem (red star) with delayed development of the floral primordia (G) that eventually bolts to produce flowers (H) with serrated floral organs and short pedicels (I-top panel, J) and cauline leaves (blue star) with ectopic disordered floral meristems (G) that develop into abnormal flowers (I-bottom panel). (K) *p35S:UFOdelFVP16* flowers with a medium *ufo-1* like phenotype. Bar = 0.1 mm (A-D, G).

After the floral transition, the cauline leaves surrounding the apical inflorescence meristem also initiated ectopic floral meristems that covered most of the leaf blade (Figure 3A–D). These floral meristems were positioned in close proximity to the axillary co-inflorescences resulting in the fasciation of the peduncle of the co-florescence and the petiole of the subtending flowering cauline leaf (Figure 3C, E).Similar to plants expressing *UFO* without *VP16*, flowers of the first group had mostly a normal organ number but the sepals were wrinkled and the petals, stamens and carpels were stunted (Figure 3F). However, the *UFO-VP16* plants with a strong phenotype belonging to the second group exhibited a more disorganized inflorescence that was delayed in development (Figure 3G). The cauline leaf primordia initiated floral meristems as observed in the first group with weaker phenotype, but remained meristematic and failed to progress into normal flowers (Figures 1S, 3I bottom panel). The flowers on the primary inflorescence were clustered due to very short pedicels and delayed internodal elongation of the peduncle (Figure 3H, I). The flowers had serrated petal-sepal chimeric organs in the first whorl and the gynoecium was either incompletely fused or replaced by additional stamens (Figure 3J). Plants transformed with *p35S:UFOdelF-VP16*, that lacked a functional F-box domain failed to produce the above described *UFO-VP16* gain-of-function phenotypes and instead showed a loss-of-function phenotype similar to *p35S:UFOdelF* without VP16 (Table S1, Figure 3K). These results further confirmed that F-box domain of UFO is required for VP16 mediated gain-of-function phenotypes.

### Meristem specification by UFO in other plant species

**Figure 4:**
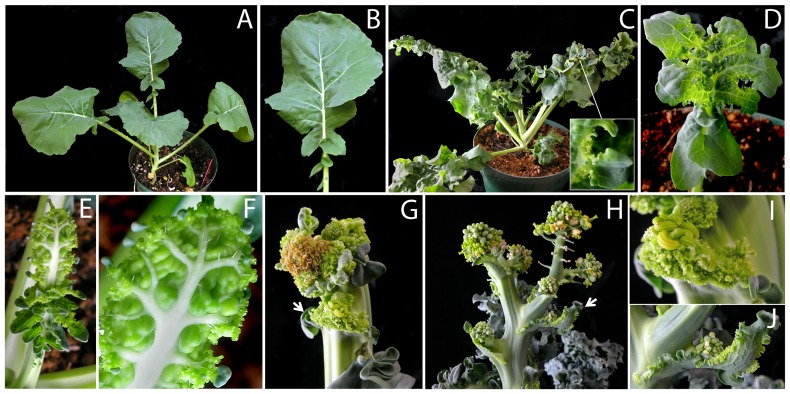
Phenotypes of *Brassica napus* plants expressing *p35S:UFO-VP16*. Wild type plant (A) showing normal lobed leaves (B). (C–F) *p35S:UFO-VP16* plant (C) showing severe lobing of the leaf (D); expanding young leaf (E) showing meristematic activity along the leaf margin (F) that leads to the enhanced lobing. Inset in (C) shows prolonged meristematic activity of the leaf margin. (G–J) Inflorescences of *p35S:UFO-VP16* plants showing severe proliferation of floral organs produced by the inflorescence meristem (G) and by the cauline leaves (G, I; white arrows); weaker phenotype showing the development of modified flowers with short pedicels (H) and prolonged meristematic activity of the cauline leaf margins (H, J).

Transformation of the related crucifer *Brassica napus* with *p35S:UFO-VP16* resulted in severe serration of the leaves in addition to the lobing observed in wild type leaves (Figure 4A–D, Table S1). These serrations were compounded as a result of reiterated serrations during leaf development (Figure 4D). Closer examination of a young leaf showed a highly meristematic leaf margin, which likely contributed to these progressively increased leaf serrations in the older leaves (Figure 4E, F). The sinus regions of these leaves often remained meristematic producing new projections for a prolonged period (Figure 4C inset). Though, expansion of the leaves was delayed, the size of the mature leaves often surpassed that of the wild type plants. Similar to Arabidopsis, the cauline leaf blade was consumed by proliferating ectopic floral structures (Figure 4G, I). The weaker *p35S:UFO-VP16* lines showed meristematic cauline leaf margins without ectopic floral meristems (Figure 4H, J). The flowers produced by the primary inflorescence in the weaker lines were clustered without sufficient elongation of the internodes and with shorter pedicels whereas in the stronger lines, a mass of proliferating floral organs was produced (Figure 4G, H). Taken together, though some differences were observed, the *p35S:UFO-VP16* mediated gain-of-function phenotypes of *B. napus* were comparable to that of Arabidopsis.

**Figure 5:**
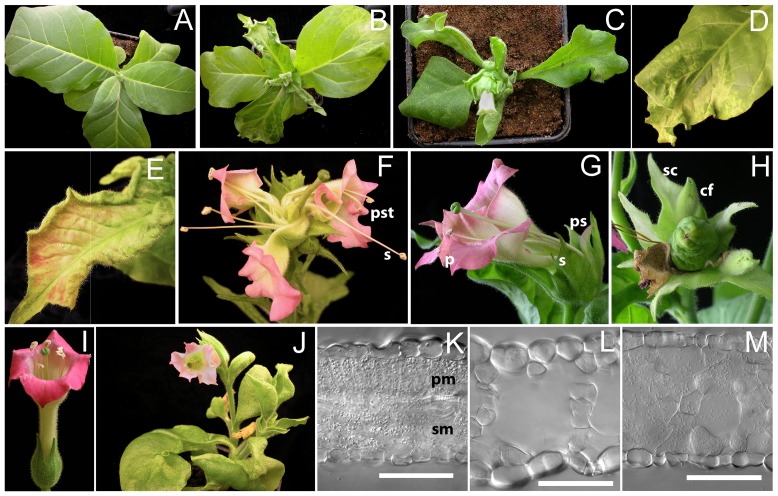
Phenotypes of tobacco plants expressing *p35S:UFO* and *p35S:UFO-VP16*. (A–C) 6 week-old tobacco plants of WT (A), *p35S:UFO* (B) and *p35S:UFO-VP16* at vegetative, bolting and flowering stages respectively. (D–H) *p35S:UFO* plant showing light green sepal-like (D) and pink petal-like (E) sectors in the vegetative leaves; flowers with spiral phyllotaxy of the sepal, petal and stamen whorls showing stamen [s], petal-stamen [pst], petal [p], petal-sepal [ps], sepal [s] and sepal-cauline leaf [sc] subtending a co-florescence [cf] (F–H); the flower in (F) shows a split corolla; the mature flower in (H) has shed its petals and stamens and shows a developing pod. (I) Wild type flower. (J) 8 week-old *p35S:UFO-VP16* showing abnormal leaves and early flowering. (K–M) Cross sections of a wild type leaf (K), a sepal (L), and a light green sector of a *p35S:UFO* vegetative leaf (M). The palisade (pm) and spongy (sm) mesophyll layers seen in the leaf are not present in the sepals and in the modified *p35S:UFO* leaf sectors. Bar = 0.1 mm.

To determine whether *UFO* could induce similar phenotypes in more unrelated plant species, the *UFO* constructs were transformed into tobacco (*Nicotiana tabacum*). In the tobacco cymose inflorescence, the apical inflorescence meristem and the consecutive co-infloresences terminate in a flower, whereas in the crucifers i.e., Arabidopsis and *B.napus*, the indeterminate racemose inflorescence produces flowers in an acropetal fashion. Tobacco plants over-expressing *UFO* produced light green sectors starting in the third leaf as well as more curling of the margins compared to wild type (Figure 5A, B; Table S1). The cellular organization of these light green sectors in the cross sections showed high similarity with sepals as opposed to leaves of wild type plants (Figure 5K–M). Pink pigmented sectors were also present occasionally in the upper leaves (Figure 5E), indicative of the chimeric nature of the vegetative leaves mixed with characteristics of sepal and petal cell types. The severity of the leaf phenotype correlated with earlier flowering, on average 57 days after seeding in *p35S:UFO* plants versus more than 90 days in wild type. The typical whorled phyllotaxy of wild type tobacco flowers was absent in the UFO over-expression lines (Figure 5F–I). Instead, the flowers exhibited a continuum of the spiral phyllotaxy of the shoot where the distinct floral whorls seen in wild type were replaced by a gradient of organ mosaics starting from cauline leaves that continued into sepal-cauline leaves, sepals, sepal-petals, petals, petal-stamens and stamens (Figure 5F-H). The *UFO-VP16* tobacco plants flowered very early (∼41 days after seeding) after producing about 5 leaves (Figure 5C); leaf curling and mosaics started in the second leaf but no ectopic floral meristems developed (Figure 5J). Taken together, *UFO* overexpression in tobacco had a distinct phenotypic effect compared to that observed in *B.napus* and Arabidopsis, highlighting the dependence of ectopic *UFO* or *UFO-VP16* on the existing developmental programs and the associated genetic factors in these species.

### UFO function depends on both LFY and SEP proteins

**Figure 6:**
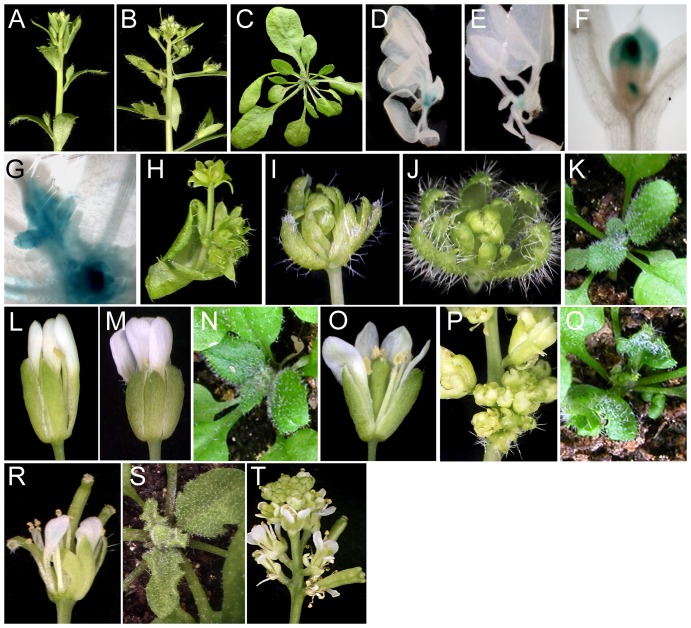
Genetic interactions between UFO and LFY, SEP. Inflorescence of *lfy-1* (A) compared to that of *lfy-1 p35S:UFO-VP16* (B). Normal rosette of *lfy-1 p35S:UFO-VP16* (C). (D, E) GUS expression of a *pLFY:GUS* in wild type (D) and *p35S:UFO-VP16* (E) seedling. (F) *pAP1:GUS p35S:UFO-VP16* seedling with GUS expression in the primary leaves. (G) *AP3:GUS p35S:UFO-VP16* shoot apex after evocation with GUS expression appearing in the leaves. (H) *ap1-10 p35S:UFO-VP16* cauline leaf with ectopic *ap1* like flowers. (I-Q) sep mutants compared with their corresponding sep UFO-VP16 transgenic lines: (I-K) *sep1 sep2-1 sep3-2 sep4-1/+*; (L–M) *sep1 SEP2-1rev/rev sep3-2/+ sep4-1*; and (O-Q) *sep1 sep2-1 sep3-2/+ SEP4*. Control flowers (I, L, O); flowers (J, M, P) and rosettes (K, N, Q) of mutants transformed with *UFO-VP16* transgene. *sep2-1* carries an En-1 insertion in the seventh intron, which has excised in the *SEP2-1rev* revertant allele. Flowers with bract-like organs (I,J) and a normal rosette (K); normal flowers (L,M) and rosette (N): normal flower (O) and *UFO-VP16* like flowers that have short pedicels with a flowering cauline leaf (arrow, P); *UFO-VP16* like rosette (Q). (R) *p35S:SEP1* inflorescence terminating in a flower. (S, T) *p35S:SEP4-VP16* rosette with leaves showing serration (S) and inflorescence producing flowers with short pedicels (T).

Floral meristems of the *lfy*-1 mutant default into a secondary co-florescence identity and are unable to develop a gain-of-function phenotype when UFO is overexpressed [7], [8]. The dependence of UFO on LFY was also observed in the present study with *UFO-VP16* as the *lfy-1* mutant was fully epistatic to the *UFO-VP16* transgene (Figure 6A–C). Expression of the *pLFY:GUS* reporter in *p35S:UFO-VP16* plants was similar to its expression in the wild type background (Figure 6D, E). Upon flowering, the GUS activity was strong in the shoot apex and leaf primordia of both lines, but was not detected in the emerging leaves. Also, *LFY* RNA levels of wild type and *p35S:UFO-VP16* seedlings were similar as determined by RT-PCR (Figure S1). These results suggest that the *LFY* expression was not upregulated by *UFO-VP16*, and the observed phenotypes were more likely the result of a prolonged interaction between UFO-VP16 and LFY during the early stages of leaf development (see discussion).

Next we tested whether *UFO-VP16* is involved in the regulation of the targets of *LFY* that include A, B, C and E class MADS-box genes [5]. Our results showed that *pAPETALA1:GUS* was expressed earlier in the lower leaves of *UFO-VP16* seedlings prior to flowering, whereas *pAPETALA3:GUS* was detected only in the upper serrated leaves at flowering stage (Figure 6F, G). No ectopic expression was observed with the *APETALA3*, *PISTILLATA* and *AGAMOUS* promoters prior to flowering (not shown). These observations are consistent with the result that none of the ABC mutants were able to suppress the serrated leaf phenotype and the flowers on both the inflorescence and the leaves retained the characteristics from the non-transformed mutants. Interestingly, serration was enhanced in the *ap1* mutant and the ectopic meristems on the leaves developed into *ap1*-like flowers (Figure 6H). These observations suggest that the ABC class MADS box genes were not required for the gain-of-function leaf phenotypes.

MADS-box proteins function in quaternary complexes to regulate the identity of the meristems and primordia in the above-ground region of the plant [28]. Generally at least one position in the complex is taken by a member of the four SEPALLATA MADS-box proteins (SEP1, SEP2, SEP3, SEP4), to mediate the formation of higher-order complexes [29]. Because the SEP proteins function in a partially redundant manner, the *UFO-VP16* construct was transformed into fertile *sep1/sep1,sep2/sep2,SEP3/sep3,SEP4/sep4* plants using the flower dip method and analyzed in the segregating offspring. The majority of 36 *sep UFO-VP16* T1 plants (24 plants) did not show leaf serration, indicating that some combinations of the *sep* alleles had suppressed the gain-of function phenotype, but instead showed flower phenotypes similar to the respective *sep* mutants without the transgene (Figure 6I–N). The second group (12 plants) showed various levels of serration, including 5 with ectopic flowers on the leaves and flowers typical for *UFO-VP16* (Figure 6O–Q). Genotyping of the segregating plants for the 4 *SEP* genes (see methods) revealed that the *sep* mutant alleles in various combinations were sufficient to suppress the UFO-VP16 phenotype of the first group. All plants with one wild type *SEP* allele and seven out of eight plants with two *SEP* alleles belonged to the first group, showing a positive correlation between the *UFO-VP16* phenotype and the presence of a higher number of wild type *SEP* alleles. Furthermore, all seven plants carrying both *SEP4* alleles had serrated leaves including five carrying ectopic flowers, indicating that SEP4 was required and is more critical than the other SEP proteins for this gain-of-function leaf phenotype. RT-PCR analysis revealed no differences in *SEP* transcript levels between *UFO-VP16* and wild type seedlings (Figure S1) suggesting that higher expression levels of the *SEP* genes were not required. Taken together, these results suggest that in addition to LFY, the SEP proteins were also required for UFO gain-of-function phenotypes in Arabidopsis.

To test whether over-activation of *SEP* could result in similar phenotypic changes as observed in *UFO* transgenic plants, *VP16* based activation constructs were developed with *SEP1* and *SEP4*. *SEP4-VP16* over-expressing plants exhibited mild serration of the leaves, which often curled up at the edges (Table S1, Figure 6S). No ectopic meristems were observed on the leaves. However the flowers were strikingly similar to those observed in *UFO-VP16* plants. The pedicels were short and the growth of the peduncle was delayed giving the inflorescence a compact appearance (Figure 6T). Further, the development of the flower buds was delayed for an extended period. Plants with a weak phenotype were taller with uneven internodal distances between the flowers. A similar weak phenotype was observed in plants transformed with *SEP1-VP16*. Both transgenics flowered at normal times, which contrasted with the very early flowering of *p35S:SEP1* transgenics and prematurely terminating in a terminal flower (Table S1, Figure 6R). Taken together, the results indicate that *UFO-VP16* and *SEP4-VP16* partly activate a similar developmental program and the dependence on LFY and SEP proteins suggest that these factors may co-regulate transcription of an overlapping group of downstream target genes.

## Discussion

### UFO functions as a co-activator in concert with LFY and SEP transcription factors

UFO encodes an F-box protein, which was shown to interact with the SCF E3 ubiquitin ligase subunits ASK1, CUL1 and subunits of the COP9 signalosome, suggesting a role in ubiquitination of proteins involved in flower development [20]. Recently LFY was shown to physically interact with UFO at the *AP3* promoter to facilitate a potential interaction between UFO and promoter elements, and further to designate LFY as an ubiquitination substrate of UFO [10], [15]. Consistent with UFO being part of a transcriptional complex, our results show that nuclear localization of UFO is required for its activity and that the Engrailed and VP16 based transcriptional modulators can modify its function. Expression of UFO lacking the F-box domain results in a loss-of-function phenotype which supports the “activation by destruction” model suggested for LFY transcription factor [10]. In this model, ubiquitination of LFY by UFO enhances its activity likely through increased recycling of LFY on the target genes' promoters, along with recruitment of the RNA polymerase II to the complex and initiation of transcription [21], [22], [23], [24]. Interestingly, the VP16 transactivation domain was unable to function in the absence of the F-box domain suggesting that the VP16 transactivator was unable to override the lack of LFY ubiquitination by UFO.

Our results also show that in addition to LFY, SEP proteins were required for the observed strong UFO-VP16 leaf serration phenotypes implying that SEP proteins may also associate with the UFO-LFY transcriptional complex. Overexpression of *SEP1* resulted in early transition of the apical meristem into a terminal flower similar to the phenotypes of plants overexpressing LFY or SEP3 [30], [31], whereas *SEP4-VP16* plants showed some phenotypic similarities with plants expressing *UFO-VP16* in the flowers. These results are consistent with the study that showed physical interaction between LFY and SEP3 proteins, and further support the model that the UFO, LFY and SEP proteins most likely interact in a transcriptional complex to regulate a common set of target genes involved in meristem specification and flower development [31].

### Function of UFO outside the APETALA3 domain

UFO has been shown to bind the *AP3* promoter in the presence of LFY to activate its expression in the stage 3 flower primordia [8], [10]. In the *ufo* mutant compared to wild type, *AP3* expression is restricted to a narrower domain in stage 4 and in later stages of flower primordia [17]. When UFO is overexpressed, the vegetative leaves become serrated, suggesting that the leaf primordia are responsive to changes in *UFO* expression [8]. Previous studies have shown that *ufo* mutants display delayed transition of the vegetative to inflorescence identity of the apical meristem. Moreover, some *ufo* flowers also lack organs in the 1^st^ and 4^th^ whorls that are established before *AP3* expression, whereas other flowers are substituted by filamentous structures [11], [12]. In the *ufo-1* mutant, *AP1* expression is reduced in stage 1 and 2 flowers [32] and consistent with this, our results show ectopic *AP1* expression in *UFO-VP16* leaves. Taken together, these phenotypes indicate that UFO in the presence of LFY is also responsible for the activation of genes other than *AP3*.

**Figure 7:**
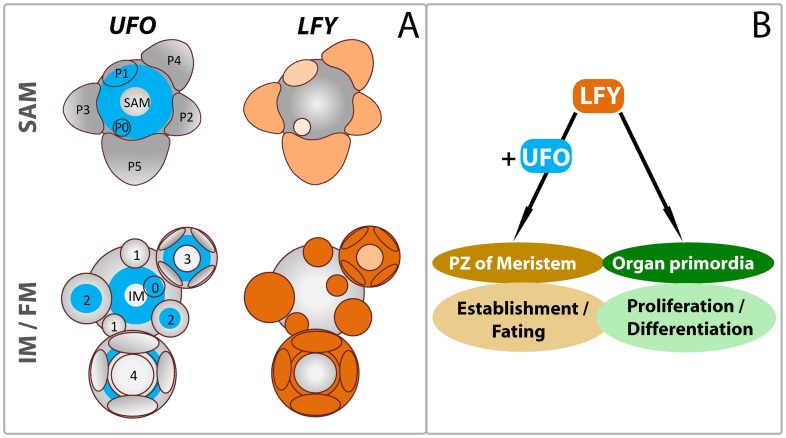
Model depicting UFO activation of LFY and their roles in the early stages of lateral meristem development. A. *UFO* (blue) is expressed in the peripheral zone of the shoot apical (SAM) and inflorescence (IM) meristems. *UFO* is later induced in the centre of stage 2 floral meristems and its expression domain is restricted to the second and third whorls during later stages [17], [34]. *LFY* expression (shades of orange) is initiated in the P0 leaf primordium and progressively increases in the later stages; its expression becoming prominent when floral meristems are initiated. The *UFO* and *LFY* expression domains overlap in the lateral P0 and P1 leaf primordia in the SAM, in stage 0 floral primordia in the IM, and in the petal, stamen and carpel whorls upon establishment [8], [36]. B. LFY activity is enhanced by UFO in the emerging lateral meristems/primordia within the peripheral zones of the meristems to ensure their establishment and fating. Lower LFY activity without UFO promotes the organogenesis of the primordia during later stages.

It was previously shown that *UFO* expression is initiated very early in the embryonic shoot meristem by *SHOOTMERISTEMLESS* (*STM*) and is maintained in the peripheral zone (PZ) of the vegetative shoot (SAM) and inflorescence (IM) meristems throughout development [8], [33], [34] (Figure 7A). In the SAM, *UFO* expression overlaps with that of *LFY* in the domains of P0 and P1 stage leaf primordia, whose expression in turn is initiated by the auxin maximum through *MONOPTEROS* (*MP*), an ARF family member of the auxin signaling pathway [35], [36]. *LFY* is expressed at very low levels in the first primary leaf primordia, but becomes increasingly stronger in the upper rosette and cauline leaf primordia [36]. In the later stages of leaf primordia, *UFO* expression is not detected whereas *LFY* continues to be expressed [34], [36] (Figure 7A). In the IM, *UFO* and *LFY* expression domains overlap in stage 0 floral primordia. *UFO* is reactivated in the centre of stage 2 flower meristems after the establishment of the first sepal whorl. When *AGAMOUS* expression is initiated in the centre during early stage 3, *UFO* RNA is restricted to a cup-shaped domain between the first and fourth whorls followed by expression at the base of petals during stage 4 while the third whorl is established [8] (Figure 7A). Taken together, *UFO* expression is not restricted to the *AP3* domain in the floral meristem, but overlaps with LFY during the early developmental stages of all lateral meristems/primordia suggesting an ancestral role in their establishment and fate specification.

### UFO is involved in the establishment of meristems

Based on the native expression patterns of *UFO* and *LFY*, we propose two functional stages for LFY activity (Figure 7B). The earlier stage which involves UFO as a co-activator is important for the lateral meristem (primordia) establishment in the PZ of the SAM, whereas the later stage of LFY function, which does not involve UFO, is primarily to promote differentiation and organogenesis of the primordia. Over-expression of *UFO* or *UFO-VP16* resulted in repeated lobing of the leaf primordia and delayed emergence in Arabidopsis (Figure 2G, H). In *B. napus*, the meristematic activity was continued in the margins of mature leaves expressing *UFO-VP16*, resulting in the production of continuous lobing and compound leaf forms (Figure 4). These results suggest that expanding the UFO expression into later stage leaf primordia prolongs the early stage function of LFY to allow continued meristem proliferation leading to serration and lobing of the leaf margins. It is interesting to note that *ufo* and *lfy* mutations do not affect the shape of the simple leaves in Arabidopsis, whereas in species with compound leaf forms, mutations in the orthologs result in a reduction of the compounded nature [37], [38], [39]. In the *ufo* mutants, LFY by itself is not sufficient to establish lateral meristems as evidenced by the reduction or lack of floral organs and the flowers, and the presence of filamentous structures [11], [12]. Additionally, the maintenance of the apical meristem is impaired, which is evident from the premature termination of the inflorescence meristem into a flower in strong *ufo* mutants. The milder *ufo* mutant phenotypes compared to those of *lfy* support the argument that UFO is not required for LFY functions in the later stages. Overexpression of *LFY* does not result in the production of serrated leaves with ectopic meristems, indicating that unlike UFO, increased LFY levels alone are not sufficient to extend the meristematic phase of the leaf primordia [30]. However, seedlings overexpressing the hyperactive LFY-VP16 form of LFY in combination with UFO lead to growth arrest, implying that higher LFY activity is important for lateral meristem establishment and that a regulated lower activity is necessary to promote growth and differentiation [9]. Overexpression of the F-box genes most similar to *UFO* (*LEAF CURLING RESPONSIVENESS* [*LCR*; At1g27340], At1g76920, At4g33160) with or without VP16 did not result in the production of aberrant phenotypes (not shown), whereas *LCR* has been implicated in the expansion of the leaf margin [40], suggesting that UFO does not function in a redundant manner with other F-box proteins.

### UFO ensures the proper fating of meristems

Phytomers are produced by the apical meristem and their fates in turn determine the identity of the vegetative, inflorescence and floral meristems [1]. The identities of the apical and lateral meristems are determined by the combinatorial co-expression of specific MADS box proteins during development [41]. LFY is responsible for activation of the MADS box genes that specify the floral meristem (*AP1, CAL, FUL*) and floral organ identities (*AP1, AP3, PI, AG, SEP*) [42]. Overexpression of *LFY* results in precocious expression of these MADS box genes leading to early flowering by homeotic transformation of the lateral shoots into single flowers followed by the premature conversion of the primary shoot into a terminal flower [30]. These overexpression *LFY* phenotypes are not *UFO* dependent. However, overexpression of *UFO-VP16* in the presence of wild type LFY results in ectopic flowers and inflorescences in the upper rosette and cauline leaves (Figure 2, 3). The arrangement of older floral meristems along the main leaf margins (Figure 2K, L) and the emergence of the new meristems in acropetal succession along the margins of the secondary leaf serrations (2L–N) are reminiscent of the acropetal development of flowers on the primary inflorescence. Furthermore, the positions of the secondary and tertiary serrations of the upper rosette leaves are indicative of a bract-like fate (Figure 2M, N) which becomes obvious in later stages of these ectopic inflorescences (Figure 2C, D). The source of the shoot-like phenotype of the leaves may originate in the axillary meristem. In Arabidopsis, immediately after emergence of the leaf primordium, a cell niche is specified in its axis for future development of the axillary meristems [43]. *REGULATOR OF AXILLARY MERISTEMS* (*RAX1*) functions to specify this axillary meristem niche and its expression is directly induced by *LFY* [44]. The fasciation of the co-inflorescences with the cauline leaves observed in *UFO-VP16* suggests that the extended meristematic phase of the leaf primordia delays the boundary formation between the axillary meristems and the leaf primordia thus allowing the co-inflorescence meristem program to expand from the axis into the leaf primordia.

Contrastingly in the *ufo* mutants, homeosis of the lateral meristems/primordia is the result of insufficient induction of *AP3* and *AP1* [17], [32], but may also apply to other genes induced by LFY [45]. In these situations, the primordia acquire an identity which was established earlier during development. For example in *ufo* flowers, petals become sepaloid and stamens carpelloid, whereas flowers are replaced by co-inflorescences or subtended by bracts. Taken together the *UFO* overexpression and *ufo* mutant phenotypes suggest that *UFO* in combination with *LFY* provides a framework for the meristems and organ primordia to establish the expression of the proper combination of identity genes, possibly by promoting the meristem fate and/or by suppressing downstream morphogenesis programs (Figure 7B).

### UFO functions in other plant species

In Arabidopsis, flowers are produced in the lateral positions of the inflorescence meristem (raceme), whereas in tobacco, flowers are positioned at the terminus of the primary and co-inflorescences (cyme), which is a function of *LFY* and *TERMINAL FLOWER1* (*TFL1*) expression in the IM [46]. Thus the flower identity in a racemose inflorescence is specified *de novo* in the lateral meristems, whereas in a cyme, the flowers are produced by a transition of the (co-) inflorescence fate into a floral identity. In tobacco, overexpression of *UFO* and *UFO-VP16* result in early flowering due to the precocious and gradual transition of the apical vegetative meristem into a terminal flower. Consistent with this interpretation, mutants of *UFO* orthologs *DOUBLE TOP* (*DOT*) and *ANANTHA* (*AN*) in the related *Solanaceae* species petunia and tomato, respectively, are unable to transit into a flower and instead reiterate the inflorescence program. The *LFY* orthologs *NFL1* (tobacco), *ABERRANT LEAF AND FLOWER* (*ALF* petunia) and *FALSIFLORA* (*FA* tomato) are expressed like *LFY* in the lateral meristems produced by the vegetative and inflorescence meristems, but the *NFL1* transcript levels are higher than those of *LFY* during the vegetative stage [39], [47], [48]. The *UFO* orthologs *DOT* in petunia and *AN* in tomato are not expressed in the vegetative meristems, but are first activated in the IMs upon transition to flowering [15], [49]. Thus in the cyme inflorescence, *DOT/AN* most likely plays an essential role to establish the floral identity in the apical meristem by activating the *LFY* orthologs.

The serrated leaves in UFO overexpressing Arabidopsis plants display some common features with *UFO/LFY* othologs in compound leaf development in other plant species. In the compound leaves of tomato the number of leaflets is slightly reduced in the *fa* mutant [39], although the majority of the leaf form is controlled by the KNOX gene pathway [50]. However, the pea *STP/UNI* pathway is important for the initiation of pinnae (i.e. leaflet and tendril) primordia in the compound leaves. *stp* (*UFO* ortholog) mutants have reduced numbers of pinnae and *uni* (*LFY* ortholog) plants have trifoliate or simple leaves [38]. Both of these mutants exhibit a prolonged meristematic phase while initiating lateral primordia in an acropetal manner and the process is reiterated in the lateral primordia upon ectopic expression of the *LFY/UNI* and/or *UFO/STP* genes [16]. Both *STP* and *UNI* are expressed in early leaf primordia and *UNI* retains expression in the distal region of the marginal blastozone until the apex terminates in a distal tendril. In the *afila* and *cochleata* mutants *UNI* is also expressed in the pinnae and stipule primordia respectively, and results in the development of secondary rachis formation in these positions demonstrating that *UNI* promotes the rachis identity of the leaf [16]. These observations raise an interesting possibility that the underpinning genetic network which increases compounding of the pea leaf by derepressing *UNI*, is similar to the reduced determinacy of the Arabidopsis leaves upon activation of *LFY* by overexpressing *UFO*. Moreover, the rachis specification of the leaf primordia by *UNI* and *STP* seems to operate in a similar fashion as the specification of the floral meristems in the inflorescence by *LFY* and *UFO* in Arabidopsis. More studies are required to address how *LFY* and *UFO* in combination with other genetic factors could produce different developmental outcomes in diverse plant species. In conclusion, we propose that the UFO orthologs play an ancestral role in activating LFY orthologs in the peripheral zone of the apical and floral meristems to promote the establishment and identity of the lateral meristems and primordia.

## Materials and Methods

### Plasmid construction

A new multiple cloning site including a C-terminal E-tag (GTTTAAACCAACTAGTAAAGATCTACAAGTTTGTACAAAGTGGTTC CGGGTGCGCCGGTGCCGTATCCGGATCCGCTGGAACCGCGTGCTCGAGCA TCGCGAGCTCTAGA) was generated by overlapping primers and cloned in the *Pme*I and *Xba*I sites of the binary Gateway destination vector pK7WG2 (VIB-Ghent University). The t35S terminator was PCR amplified with primers CACCTCGCGATGACGGCCATGCTAGAGTCCGCA and TCTAGAGTCACTGGATTTTGGTTTTAGG, and cloned as an *Nru*I/*Xba*I fragment in the respective sites of the new MCS. The *Bsr*GI Gateway cassette (GW) fragment and the *Pme*I/*Spe*I p35S promoter fragment were reintroduced from pK7WG2 by cloning in the respective sites of the new MCS resulting in pER310. The engrailed repressor domain was PCR amplified from pLD16125 (Drosophila Genomics Resource Centre) with primers CACCACTAGTATGGCCCTGGAGGATCGCTG and AGATCTGGATCCCAGAGCAGATTTCTC and inserted at the N-terminal side of the GW site in pER310 resulting in pER311. The glucocorticoid receptor was amplified with primers CACCCTCGAGCAAAGAAAAAAATCAAAGGGATTC and TCGCGATCATTTTTGATGAAACAGAAG and cloned in frame downstream from the E-tag in pER310 leading to pER312. Likewise in pER430 the VP16 domain was inserted after amplification with primers CACCGCTCGAGCCCCCCCGACCGATGTCAGCCTG and TCGCGATCACCCACCGTACTCGTCAAT. YFP was amplified with primers CACCACTAGTCGACTTATGGTGAGCAAGGGCGAGGA and TCGCGATTAGGATCCCTTATACAGCTCGTCCATGCC and cloned C-terminal of the E-tag in pER310 resulting in pER562. The coding sequences without stop codon of *UFO*, *UFOdelF* (codons 50–62 deleted), *SEP1*, *SEP4*, and *GUS* were cloned in pDONR201 (Invitrogen) before recombination in the designated T-DNA destination vectors. The three-way pER557 was created by substituting the p35S promoter and attR1 fragment of pER310 with the attR4 recombination site. The promoters including the start codon of the Arabidopsis genes *LFY* (-2321), *AP1* (-1455), *AP3* (-1240), *PI* (-960) and *AG* (-563-codon 83) were cloned into pDONR P4-P1R (invitrogen) and combined with GUS or other genes in pER557 using LR clonase.

### Plant materials, transformation and genotyping

Mutants used in this study: *sgs2-1* [25], *sep1,2,3,4* [51], *lfy-1, ufo-1, ask1-1, ap1-10, pi-1, ap3-1* and *ag* were provided by the Arabidopsis Biological Resource Center (ABRC, Ohio). Plants were grown at 22°C, 4000 lux under long day (16 h light) or short day (10 h light) regimes. Constructs were transformed in wild type Arabidopsis thaliana ecotype Columbia or in mutants with their designated background using the Agrobacterium floral dip method [52]. Transgenic plants were selected on ½ MS medium supplemented with 10 g/L sucrose and 10 mg/L L-PPT or 50 mg/L kanamycin, and transplanted in soil after seven days. UFO-GR plants were induced by spraying with 30 µM dexamethasone, 0.01% Tween-20 in water. Genotyping of the SEP alleles was performed as described by Ditta et al. [51]. *Brassica napus* DH1250 hypocotyls and *Nicotiana tabacum* Xanthi leaf segments were used for Agrobacterium mediated transformation. The number of transgenic lines analyzed in this study is summarized in Table S1. Flowering time of the transgenic tobacco lines was compared with wild type after seeding T1 seed in soil and after selection for the transgene by spraying with 100 mg/L L-PPT + 50 µl/L Silwet.

### qRT-PCR

The *UFO*, *LFY*, *SEP1*, *2*, and *3* transcript levels and relative expression levels were quantified by qRT-PCR using *actin 2* as internal controls using the methods described in [53]. The qRT-PCR experiment was performed in triplicate on 5 pools of 10 day-old T1 seedlings of *35S:UFO-VP16* and Col WT using the Applied Biosystem Step One real-time PCR system and the SYBR Green PCR master mix as detailed in [53]. The tissue used for qRT-PCR included the SAM and surrounding primary leaves. The primers used for qRT-PCR are listed in Table S2.

### GUS staining and microscopy

Plant tissue was vacuum infiltrated in a solution containing 100 mM NaHPO4 pH 7.0, 10 mM Na-EDTA, 5 mM Na-ferricyanide, 5 mM K-ferrocyanide, 0.1% Triton X-100, 1 mg/ml 5-bromo-4 chloro 3 indolyl β-D-glucuronide (X-GlcU) and incubated overnight at 37°C. Tissue was cleared in 95% ethanol. Plant samples for scanning electron microscopy (SEM) were fixed overnight in 3% glutaraldehyde in 25 mM NaHPO4 buffer pH 7.0 at 4°C. After rinsing with the same phosphate buffer, samples were transferred to PO4 buffer with 1% OsO4 and incubated for 2 hours at room temperature. After rinsing with buffer the samples were transferred to 100% acetone with 10% increments. After two more changes with acetone the samples were critical point dried, mounted on aluminum stubs and coated with gold for 3 minutes in an Edwards S150B sputter coater. The samples were observed under a Phillips 505 scanning electron microscope at 30 kV and images were captured on Polaroid film. Images were scanned and edited in Adobe Photoshop CS (Adobe Systems, San Jose, California) to improve the contrast and place scale bars.

## Supporting Information

Figure S1 **RT-PCR results of gene expression levels in p35S:UFO-VP16 seedlings compared to wild type Arabidopsis.**
(TIF)

Table S1 **Summary of** ***UFO*** **transgenic lines used in this study showing the distribution of the various phenotypes.**
(DOCX)

Table S2 **Primers used for RT-PCR analysis.**
(DOCX)

## References

[1] Chandler JW (2012) Floral meristem initiation and emergence in plants. Cell Mol Life Sci 69: 3807–3818. 22573183 10.1007/s00018-012-0999-0 PMC11115123

[2] Barton MK (2010) Twenty years on: The inner workings of the shoot apical meristem, a developmental dynamo. Dev Biol 341: 95–113. 19961843 10.1016/j.ydbio.2009.11.029

[3] Efroni I, Eshed Y, Lifschitz E (2010) Morphogenesis of Simple and Compound Leaves: A Critical Review. Plant Cell 22: 1019–1032. 20435903 10.1105/tpc.109.073601 PMC2879760

[4] Wu S, Smith HS (2012) Out of step: The function of TALE homeodomain transcription factors that regulate shoot meristem maintenance and meristem identity. Frontiers in Biology 7: 144–154.

[5] Maizel A, Busch MA, Tanahashi T, Perkovic J, Kato M, et al (2005) The floral regulator LEAFY evolves by substitutions in the DNA binding domain. Science 308: 260–263. 15821093 10.1126/science.1108229

[6] Huala E, Sussex IM (1992) LEAFY Interacts with Floral Homeotic Genes to Regulate Arabidopsis Floral Development. Plant Cell 4: 901–913. 12297664 10.1105/tpc.4.8.901 PMC160183

[7] Weigel D, Alvarez J, Smyth DR, Yanofsky MF, Meyerowitz EM (1992) LEAFY controls floral meristem identity in Arabidopsis. Cell 69: 843–859. 1350515 10.1016/0092-8674(92)90295-n

[8] Lee I, Wolfe DS, Nilsson O, Weigel D (1997) A LEAFY co-regulator encoded by UNUSUAL FLORAL ORGANS. Curr Biol 7: 95–104. 9016705 10.1016/s0960-9822(06)00053-4

[9] Parcy F, Nilsson O, Busch MA, Lee I, Weigel D (1998) A genetic framework for floral patterning. Nature 395: 561–566. 9783581 10.1038/26903

[10] Chae E, Tan QK, Hill TA, Irish VF (2008) An Arabidopsis F-box protein acts as a transcriptional co-factor to regulate floral development. Development 135: 1235–1245. 18287201 10.1242/dev.015842

[11] Levin JZ, Meyerowitz EM (1995) UFO: An Arabidopsis Gene Involved in Both Floral Meristem and Floral Organ Development. Plant Cell 7: 529–548. 7780306 10.1105/tpc.7.5.529 PMC160802

[12] Wilkinson MD, Haughn GW (1995) UNUSUAL FLORAL ORGANS Controls Meristem Identity and Organ Primordia Fate in Arabidopsis. Plant Cell 7: 1485–1499. 12242408 10.1105/tpc.7.9.1485 PMC160975

[13] McKim S, Hay A (2010) Patterning and evolution of floral structures - marking time. Curr Opin Genet Dev 20: 448–453. 20452201 10.1016/j.gde.2010.04.007

[14] Moyroud E, Kusters E, Monniaux M, Koes R, Parcy F (2010) LEAFY blossoms. Trends Plant Sci 15: 346–352. 20413341 10.1016/j.tplants.2010.03.007

[15] Souer E, Rebocho AB, Bliek M, Kusters E, de Bruin RA, et al (2008) Patterning of inflorescences and flowers by the F-Box protein DOUBLE TOP and the LEAFY homolog ABERRANT LEAF AND FLOWER of petunia. Plant Cell 20: 2033–2048. 18713949 10.1105/tpc.108.060871 PMC2553618

[16] Gourlay CW, Hofer JM, Ellis TH (2000) Pea compound leaf architecture is regulated by interactions among the genes UNIFOLIATA, cochleata, afila, and tendril-lessn. Plant Cell 12: 1279–1294. 10948249 10.1105/tpc.12.8.1279 PMC149102

[17] Samach A, Klenz JE, Kohalmi SE, Risseeuw E, Haughn GW, et al (1999) The UNUSUAL FLORAL ORGANS gene of Arabidopsis thaliana is an F-box protein required for normal patterning and growth in the floral meristem. Plant J 20: 433–445. 10607296 10.1046/j.1365-313x.1999.00617.x

[18] Risseeuw EP, Daskalchuk TE, Banks TW, Liu E, Cotelesage J, et al (2003) Protein interaction analysis of SCF ubiquitin E3 ligase subunits from Arabidopsis. Plant J 34: 753–767. 12795696 10.1046/j.1365-313x.2003.01768.x

[19] Lechner E, Achard P, Vansiri A, Potuschak T, Genschik P (2006) F-box proteins everywhere. Curr Opin Plant Biol 9: 631–638. 17005440 10.1016/j.pbi.2006.09.003

[20] Wang X, Feng S, Nakayama N, Crosby WL, Irish V, et al (2003) The COP9 signalosome interacts with SCF UFO and participates in Arabidopsis flower development. Plant Cell 15: 1071–1082. 12724534 10.1105/tpc.009936 PMC153717

[21] Salghetti SE, Caudy AA, Chenoweth JG, Tansey WP (2001) Regulation of transcriptional activation domain function by ubiquitin. Science 293: 1651–1653. 11463878 10.1126/science.1062079

[22] Kim SY, Herbst A, Tworkowski KA, Salghetti SE, Tansey WP (2003) Skp2 regulates Myc protein stability and activity. Mol Cell 11: 1177–1188. 12769843 10.1016/s1097-2765(03)00173-4

[23] Lipford JR, Smith GT, Chi Y, Deshaies RJ (2005) A putative stimulatory role for activator turnover in gene expression. Nature 438: 113–116. 16267558 10.1038/nature04098

[24] von der Lehr N, Johansson S, Wu S, Bahram F, Castell A, et al (2003) The F-Box Protein Skp2 Participates in c-Myc Proteosomal Degradation and Acts as a Cofactor for c-Myc-Regulated Transcription. Mol Cell 11: 1189–1200. 12769844 10.1016/s1097-2765(03)00193-x

[25] Mourrain P, Beclin C, Elmayan T, Feuerbach F, Godon C, et al (2000) Arabidopsis SGS2 and SGS3 genes are required for posttranscriptional gene silencing and natural virus resistance. Cell 101: 533–542. 10850495 10.1016/s0092-8674(00)80863-6

[26] Zhao D, Yu Q, Chen M, Ma H (2001) The ASK1 gene regulates B function gene expression in cooperation with UFO and LEAFY in Arabidopsis. Development 128: 2735–2746. 11526079 10.1242/dev.128.14.2735

[27] Smyth DR, Bowman JL, Meyerowitz EM (1990) Early flower development in Arabidopsis. Plant Cell 2: 755–767. 2152125 10.1105/tpc.2.8.755 PMC159928

[28] Honma T, Goto K (2001) Complexes of MADS-box proteins are sufficient to convert leaves into floral organs. Nature 409: 525–529. 11206550 10.1038/35054083

[29] Smaczniak C, Immink RGH, Muiño JM, Blanvillain R, Busscher M, et al.. (2012) Characterization of MADS-domain transcription factor complexes in Arabidopsis flower development. Proc Natl Acad Sci U S A. 10.1073/pnas.1112871109 PMC3277181 22238427

[30] Weigel D, Nilsson O (1995) A developmental switch sufficient for flower initiation in diverse plants. Nature 377: 495–500. 7566146 10.1038/377495a0

[31] Castillejo C, Romera-Branchat M, Pelaz S (2005) A new role of the Arabidopsis SEPALLATA3 gene revealed by its constitutive expression. Plant J 43: 586–596. 16098111 10.1111/j.1365-313X.2005.02476.x

[32] Hepworth SR, Klenz JE, Haughn GW (2006) UFO in the Arabidopsis inflorescence apex is required for floral-meristem identity and bract suppression. Planta 223: 769–778. 16244866 10.1007/s00425-005-0138-3

[33] Long JA, Barton MK (1998) The development of apical embryonic pattern in Arabidopsis. Development 125: 3027–3035. 9671577 10.1242/dev.125.16.3027

[34] Reddy GV (2008) Live-imaging stem-cell homeostasis in the Arabidopsis shoot apex. Curr Opin Plant Biol 11: 88–93. 18069047 10.1016/j.pbi.2007.10.012

[35] Yamaguchi N, Wu M-F, Winter Cara M, Berns Markus C, Nole-Wilson S, et al (2013) A Molecular Framework for Auxin-Mediated Initiation of Flower Primordia. Dev Cell 24: 271–282. 23375585 10.1016/j.devcel.2012.12.017

[36] Blazquez MA, Soowal LN, Lee I, Weigel D (1997) LEAFY expression and flower initiation in Arabidopsis. Development 124: 3835–3844. 9367439 10.1242/dev.124.19.3835

[37] Hofer J, Turner L, Moreau C, Ambrose M, Isaac P, et al (2009) Tendril-less regulates tendril formation in pea leaves. Plant Cell 21: 420–428. 19208900 10.1105/tpc.108.064071 PMC2660626

[38] Taylor S, Hofer J, Murfet I (2001) Stamina pistilloida, the Pea Ortholog of Fim and UFO, Is Required for Normal Development of Flowers, Inflorescences, and Leaves. Plant Cell 13: 31–46. 11158527 10.1105/tpc.13.1.31 PMC102211

[39] Molinero-Rosales N, Jamilena M, Zurita S, Gómez P, Capel J, et al (1999) FALSIFLORA, the tomato orthologue of FLORICAULA and LEAFY, controls flowering time and floral meristem identity. Plant J 20: 685–693. 10652140 10.1046/j.1365-313x.1999.00641.x

[40] Song JB, Huang SQ, Dalmay T, Yang ZM (2012) Regulation of Leaf Morphology by MicroRNA394 and its Target LEAF CURLING RESPONSIVENESS. Plant Cell Physiol 53: 1283–1294. 22619471 10.1093/pcp/pcs080

[41] Smaczniak C, Immink RGH, Angenent GC, Kaufmann K (2012) Developmental and evolutionary diversity of plant MADS-domain factors: insights from recent studies. Development 139: 3081–3098. 22872082 10.1242/dev.074674

[42] Kaufmann K, Pajoro A, Angenent GC (2010) Regulation of transcription in plants: mechanisms controlling developmental switches. Nat Rev Genet 11: 830–842. 21063441 10.1038/nrg2885

[43] Keller T, Abbott J, Moritz T, Doerner P (2006) Arabidopsis REGULATOR OF AXILLARY MERISTEMS1 Controls a Leaf Axil Stem Cell Niche and Modulates Vegetative Development. Plant Cell 18: 598–611. 16473968 10.1105/tpc.105.038588 PMC1383636

[44] Chahtane H, Vachon G, Le Masson M, Thévenon E, Périgon S, et al (2013) A variant of LEAFY reveals its capacity to stimulate meristem development by inducing RAX1. Plant J 74: 678–689. 23445516 10.1111/tpj.12156

[45] Siriwardana NS, Lamb RS (2012) The poetry of reproduction: the role of LEAFY in Arabidopsis thaliana flower formation. Int J Dev Biol 56: 207–221. 22451042 10.1387/ijdb.113450ns

[46] Prusinkiewicz P, Erasmus Y, Lane B, Harder LD, Coen E (2007) Evolution and development of inflorescence architectures. Science 316: 1452–1456. 17525303 10.1126/science.1140429

[47] Kelly AJ, Bonnlander MB, Meeks-Wagner DR (1995) NFL, the tobacco homolog of FLORICAULA and LEAFY, is transcriptionally expressed in both vegetative and floral meristems. Plant Cell 7: 225–234. 7756832 10.1105/tpc.7.2.225 PMC160778

[48] Souer E, van der Krol A, Kloos D, Spelt C, Bliek M, et al (1998) Genetic control of branching pattern and floral identity during Petunia inflorescence development. Development 125: 733–742. 9435293 10.1242/dev.125.4.733

[49] Lippman ZB, Cohen O, Alvarez JP, Abu-Abied M, Pekker I, et al (2008) The making of a compound inflorescence in tomato and related nightshades. PLoS Biol 6: e288. 19018664 10.1371/journal.pbio.0060288 PMC2586368

[50] Bharathan G, Goliber TE, Moore C, Kessler S, Pham T, et al (2002) Homologies in leaf form inferred from KNOXI gene expression during development. Science 296: 1858–1860. 12052958 10.1126/science.1070343

[51] Ditta G, Pinyopich A, Robles P, Pelaz S, Yanofsky MF (2004) The SEP4 gene of Arabidopsis thaliana functions in floral organ and meristem identity. Curr Biol 14: 1935–1940. 15530395 10.1016/j.cub.2004.10.028

[52] Clough SJ, Bent AF (1998) Floral dip: a simplified method forAgrobacterium-mediated transformation ofArabidopsis thaliana. Plant J 16: 735–743. 10069079 10.1046/j.1365-313x.1998.00343.x

[53] Xiang D, Yang H, Venglat P, Cao Y, Wen R, et al (2011) POPCORN functions in the auxin pathway to regulate embryonic body plan and meristem organization in Arabidopsis. Plant Cell 23: 4348–4367. 22158464 10.1105/tpc.111.091777 PMC3269870

