# Supplementary figures and images for: An Activated Form of UFO Alters Leaf Development and Produces Ectopic Floral and Inflorescence Meristems
Source: PLoS One. 2013 Dec 23;8(12):e83807. doi: 10.1371/journal.pone.0083807 (PMC3871548; doi:10.1371/journal.pone.0083807)

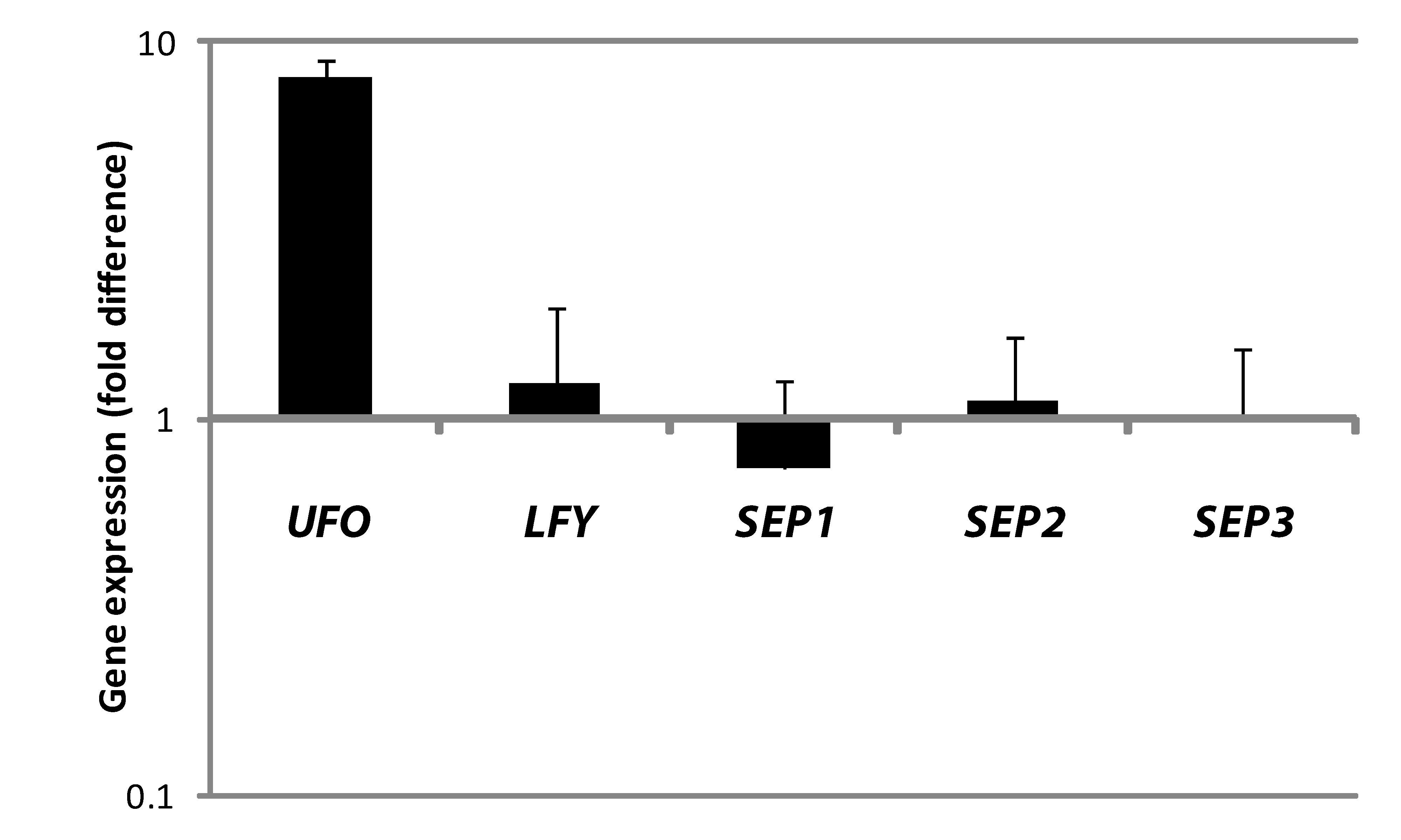

Supplement: Figure S1 — RT-PCR results of gene expression levels in p35S:UFO-VP16 seedlings compared to wild type Arabidopsis. (TIF) [file pone.0083807.s001.tif]
