# Supplementary material for: An Activated Form of UFO Alters Leaf Development and Produces Ectopic Floral and Inflorescence Meristems
Source: PLoS One. 2013 Dec 23;8(12):e83807. doi: 10.1371/journal.pone.0083807 (PMC3871548; doi:10.1371/journal.pone.0083807)
Supplement: Table S1 — Summary of UFO transgenic lines used in this study showing the distribution of the various phenotypes. (DOCX) [file pone.0083807.s002.docx]

**Table S1.** Summary of *UFO* transgenic lines used in this study showing the distribution of the various phenotypes.

| **Genotypes** | **# lines** | **Gain-of-function %** | | | **Loss-of-function %** | | | **WT-like %** |
| --- | --- | --- | --- | --- | --- | --- | --- | --- |
| ***Arabidopsis*** |  | **W** | **M** | **S** | **W** | **M** | **S** |  |
| ***p35S:UFO*** | 51 | 22 |  |  | 30 | 35 |  | 14 |
| ***sgs2-1 p35S:UFO*** | 53 | 98 |  |  |  |  |  | 2 |
| ***p35S:UFO-GR (-)*** | 30 |  |  |  | 37 | 13 |  | 50 |
| ***p35S:UFO-GR (+)*** | 26 | 23 |  |  | 46 | 8 |  | 23 |
| ***pLFY:UFO*** | 47 | 4 |  |  | 11 | 6 |  | 79 |
| ***UFOdelF*** | 45 |  |  |  | 24 | 53 | 7 | 16 |
| ***p35S:En-UFO*** | 48 |  |  |  | 15 | 54 | 17 | 15 |
| ***p35S:En-UFOdelF*** | 20 |  |  |  |  | 65 | 20 | 15 |
| ***p35S:UFO-VP16*** | 28 | 36 | 50 | 14 |  |  |  |  |
| ***sgs2-1 p35S:UFO-VP16*** | 52 |  |  | 100 |  |  |  |  |
| ***p35S:UFOdelF-VP16*** | 51 |  |  |  | 16 | 41 |  | 43 |
| ***p35S:SEP1*** | 53 |  | 21 |  |  |  |  | 79 |
| ***p35S:SEP1-VP16*** | 53 | 42 | 4 |  |  |  |  | 54 |
| ***p35S:SEP4-VP16*** | 48 | 27 | 50 |  |  |  |  | 23 |
| ***Brassica napus*** | **# lines** | **Gain-of-function %** | | |  |  |  | **WT-like %** |
|  |  | **W** |  | **S** |  |  |  |  |
| ***p35S:UFO-VP16*** | 36 | 14 |  | 11 |  |  |  | 75 |
| ***Nicotiana tabacum*** | **# lines** | **Gain-of-function %** | | |  |  |  | **WT-like %** |
|  |  | **W** |  | **S** |  |  |  |  |
| ***p35S:UFO*** | 18 | 66 |  |  |  |  |  | 34 |
| ***p35S:UFO-VP16*** | 14 |  |  | 48 |  |  |  | 42 |

W: weak, M: medium, S: strong phenotypes

Description of *Arabidopsis* UFO phenotypes:

Gain-of-function: W- serrated leaves and split / short gynoecia; M- serrated leaves with ectopic flowers; S- severely serrated leaves with ectopic meristems, serrated floral organs and short pedicels.

SEP phenotypes and phenotypes for *B. napus* and *N. tabacum* are described in the text.

Loss-of-function: W- occasional missing petals; M- frequent missing petals and petal-stamen chimera; S- *ufo-1* like phenotype with petal and stamen reduction to filaments.
