# Supplementary material for: An Activated Form of UFO Alters Leaf Development and Produces Ectopic Floral and Inflorescence Meristems
Source: PLoS One. 2013 Dec 23;8(12):e83807. doi: 10.1371/journal.pone.0083807 (PMC3871548; doi:10.1371/journal.pone.0083807)
Supplement: Table S2 — Primers used for RT-PCR analysis. (DOCX) [file pone.0083807.s003.docx]

**Table S2:** Primers used for RT-PCR analysis

| **Gene** | **Forward primer** | **Reverse primer** |
| --- | --- | --- |
| **UFO** | 5’- ACGGCTTTGTAGCTTGGAAT-3’ | 5’- TCATCGGAGCTTGAATCTTG-3’ |
| **LFY** | 5`-GAACAATGCCGTGAGTTCCT-3’ | 5`-TCGCTCCTGATTTCTTCGCG-3’ |
| **SEP1** | 5`-AGGAGGATGGGAAGGTGGTG-3’ | 5`-GAGCTTGGGTTGTCGCAGTT-3’ |
| **SEP2** | 5`-AGGATGGGAAGGTGGTGATC-3’ | 5`-GACCTTGCACCGTCACAGCC-3’ |
| **SEP3** | 5`-CTAACCAAGAAGAGGTTGATC-3’ | 5`-TCACACTTGGTCCTGCTCCC-3’ |
| **SEP4** | 5`-CAACAACAACATCAACAACAGC-3’ | 5`-GAGTTGGTTGCATTTGCAGG-3’ |
| **ACT2** | 5’-TCCCTCAGCACATTCCAGCAGAT-3’ | 5’-AACGATTCCTGGACCTGCCTCATC-3’ |
